# Cocoa shell extract supplementation in hypertensive rats induces outward hypertrophic remodelling in mesenteric resistance arteries, reducing fibrosis

**DOI:** 10.1113/EP092528

**Published:** 2025-05-05

**Authors:** Pilar Rodríguez‐Rodríguez, Santiago Ruvira, Fatima Abderrahim, Dolores Morales, Metee Iampanichakul, Lucía Juárez, David Ramiro‐Cortijo, Silvia M. Arribas

**Affiliations:** ^1^ Department of Physiology, Faculty of Medicine Universidad Autónoma de Madrid Madrid Spain; ^2^ Food, Oxidative Stress and Cardiovascular Health (FOSCH) Research Group Universidad Autónoma de Madrid Madrid Spain; ^3^ Confocal Microscopy Service (SiDI), Faculty of Medicine Universidad Autónoma de Madrid Madrid Spain; ^4^ Department of Physiology, Faculty of Medicine Khon Kaen University Khon Kaen Thailand

**Keywords:** cocoa shell extract, collagen, fetal programming, hypertension, remodelling, resistance arteries, stiffness

## Abstract

Hypertension is the leading risk factor for cardiovascular diseases and is difficult to control, putting the focus on foods and nutraceuticals. Cocoa has beneficial cardiovascular effects but generates large amounts of residues, such as cocoa shells, which contain bioactive molecules. In a rat model of hypertension (in males) induced by fetal–maternal undernutrition (MUN), supplementation with cocoa shell extract (CSE) at 250 mg/kg for 3 weeks reduced blood pressure, improving mesenteric resistance artery (MRA) vasodilatation. We aimed to investigate, in the same rats, the effect of supplementation on MRA remodelling and stiffening, assessing modifications in cellular and extracellular matrix and contractility. Structural and mechanical parameters were studied with pressure myography, vasoconstriction with wire myography, and cells, collagen and internal elastic lamina fenestrae with confocal microscopy. In comparison to sex‐matched control animals, MUN males and females showed a reduction in MRA diameter. However, only MUN males exhibited stiffening, reduced media and increased adventitial layer thickness, and augmented collagen content and noradrenaline‐induced contractions. CSE induced outward hypertrophic remodelling, with a normal wall‐to‐lumen ratio in all groups, and a hypertrophic effect on smooth muscle cells. In addition, in MUN males CSE reduced MRA adventitial collagen and improved elasticity. In conclusion, CSE supplementation induces MRA outward hypertrophic remodelling, possibly related to improved flood flow. In MUN hypertensive rats, the increased diameter and elasticity, associated with modification to the ratio of cells to extracellular matrix, might contribute to the blood pressure‐lowering effects of CSE.

## Abstract (highlights)

- **What is the central question of this study?**
  Are antihypertensive effects of cocoa shell extract (CSE) related to its capacity to reverse remodelling and stiffening of mesenteric resistance arteries (MRA) in the rat fetal–maternal undernutrition model of programmed hypertension?
- **What is the main finding and its importance?**
  CSE supplementation induced outward remodelling with medial hypertrophy in MRA, independent of sex and group. In hypertensive male rats with MRA inward remodelling and fibrosis, CSE supplementation reduced stiffness through modifications in collagen and elastin. Remodelling induced by CSE could be a consequence of increased blood flow owing to improved dilatation, previously demonstrated in MRA. Structural and mechanical modifications might contribute to blood pressure‐lowering effects of CSE supplementation.

## 1 INTRODUCTION

Cardiovascular diseases remain the leading cause of death worldwide, with a high socioeconomic burden, and arterial hypertension is one of the main risk factors (Vaduganathan et al., 2022). Hypertension has a multifactorial origin and, together with genetic background and lifestyle, exposure during the intrauterine period to stress factors affecting fetal development also plays a key role (Penkler et al., 2019). Among them, restriction of nutrient access to the fetus, and subsequent low birth weight, stands out, as demonstrated in descendants from pregnant women suffering food deprivation owing to war or famine (Torreggiani et al., 2024), or with obstetric complications leading to uteroplacental insufficiency and intrauterine growth restriction (Chatmethakul & Roghair, 2019; Sehgal et al., 2023). Overall, low birth weight affects a large number of pregnancies worldwide, and is a relevant cardiovascular risk factor.

Hypertension is associated with resistance artery remodelling (Feihl et al., 2008; Ma et al., 2023), characterized by lumen narrowing and stiffness related to extracellular matrix (ECM) alterations, such as excess collagen or fragmentation of elastic fibres (Ma et al., 2023; Mulvany, 2012; Schiffrin, 2004). Vascular structural alterations perpetuate hypertension and also contribute to adverse cardiovascular events (Rizzoni et al., 2003) and organ damage, being a sign of risk of disease progression (Masi et al., 2020). Therefore, antihypertensive drugs with the capacity to reverse remodelling and stiffening are of interest (Boutouyrie et al., 2021; Brown et al., 2018). Adverse effects and the need for two or more drug classes to reduce blood pressure effectively (Thomopoulos et al., 2016) have turned attention to foods and natural products containing bioactive components with cardiovascular protective actions. Among them, cocoa (*Theobroma cacao* L.) is of interest, with positive results in intervention trials demonstrating that consumption of dark chocolate can reduce cardiovascular risk (Arisi et al., 2024; Goya et al., 2022; Martin & Ramos, 2021). The cocoa food industry generates many inedible by‐products, such as the shell or husk, mostly discarded as waste that contributes to environmental pollution. Its high content of bioactive compounds makes it suitable for health applications (Soares & Oliveira, 2022; Younes et al., 2023). From this matrix, we have obtained an aqueous extract [cocoa shell extract (CSE)] enriched in polyphenols and methylxanthines (Rebollo‐Hernanz et al., 2021) with antioxidant actions in vitro (Cañas et al., 2023) and ex vivo vasodilatory effects (Rodríguez‐Rodríguez et al., 2022). In a rat model of hypertension induced by maternal undernutrition (MUN rats), we have also demonstrated that supplementation with CSE has blood pressure‐lowering effects (Ruvira et al., 2024). The aim of the present study was to evaluate whether the antihypertensive effects of CSE are related to its capacity to reverse the remodelling and stiffening of resistance arteries in the MUN model of programmed hypertension.

## 2 MATERIALS AND METHODS

### 2.1 Ethical approval

Sprague–Dawley adult rats procured from the Animal House facility of the Universidad Autónoma de Madrid (UAM; ES‐28079‐0000097) were used for this study. The experiments were approved by the Ethics Review Boards of UAM (CEI‐UAM 96‐1776‐A286) and the Regional Environment Committee of the Comunidad Autónoma de Madrid (RD 53/2013; reference PROEX 04/19). The experiments conformed to the *Guidelines for the Care and Use of Laboratory Animals* (US National Institutes of Health publication no. 85‐23, revised in 1996), the EU Directive 2010/63/EU on the protection of animals, and the Spanish legislation (RD53/2013).

The rats were kept with a 12 h–12 h light–dark photoperiod and were maintained at constant temperature (22°C) and relative humidity (40%). Adult rats of the same sex were housed in buckets (36.5 cm/21.5 cm/18.5 cm length/width/height) on aspen wood bedding and fed with a standard breeding diet (Euro Rodent Diet 22; 5LF5, Labdiet, Madrid, Spain; 55% carbohydrates, 22% protein, 4.4% fat, 4.1% fibre and 5.4% mineral), with water provided ad libitum. Animal health and welfare were monitored regularly by the Animal House Facility staff.

### 2.2 Animal model and supplementation

The model of fetal programming of hypertension was induced by rat undernutrition during the second part of gestation, as previously described (Rodríguez‐Rodríguez et al., 2023). Briefly, after mating, day 1 of gestation was established by the presence of a vaginal plug. The rats were then allocated to control (*n* = 3 dams) or MUN (*n* = 3 dams) groups. MUN rats were fed ad libitum during the first 10 days and with 12 g/day (this amount was previously calculated as 50% of the usual rat daily intake) until delivery, returning to ad libitum during lactation. Control dams received food ad libitum throughout gestation and lactation. At birth, the litter size was established as 12 individuals, 6 females and 6 males, whenever possible. The rats were weaned at the age of 21 days, and the males and females were maintained in different cages of three to five individuals (according to weight) until 8–10 months of age, when experimental procedures were initiated, using two females and two males from each litter per group.

Supplementation was based on voluntary acceptance of a gelatine cube as previously described (Ruvira et al., 2024, 2023). The cubes were prepared in moulds, with neutral gelatine (Inkafoods, S.L., Barcelona, Spain) in hot water at a concentration of 140 g/L, with flavouring and sweetener (VEH); CSE cubes were prepared with addition of the extract at 250 mg/kg, previously calculated according to the rat weight. The rats were first trained with a VEH gelatine cube and, once complete ingestion was observed, supplementation started. CSE or VEH supplementation was given 5 days per week for 3 weeks. We established four experimental groups, including six males and six females from three different litters per group: control rats supplemented with vehicle (Control‐VEH); control rats supplemented with CSE (Control‐CSE); MUN rats supplemented with vehicle (MUN‐VEH); and MUN rats supplemented with CSE (MUN‐CSE).

### 2.3 Protocol design and experimental procedures

In the present work, we used the same rats as in our previous study, in which blood pressure was analysed by tail‐cuff plethysmography. We established that MUN males (but not MUN females) were hypertensive and that CSE supplementation significantly reduced blood pressure in MUN males, without an effect in other groups (Ruvira et al., 2024). After blood pressure evaluation, the rats were killed by increasing CO_2_ inhalation, followed by exsanguination. All procedures were performed according to the rules of the Ethical Committees of the University and Comunidad Autónoma de Madrid, and the ARRIVE guidelines (https://physoc.onlinelibrary.wiley.com/hub/animal‐experiments) (Percie du Sert et al., 2020).

**FIGURE 1:**
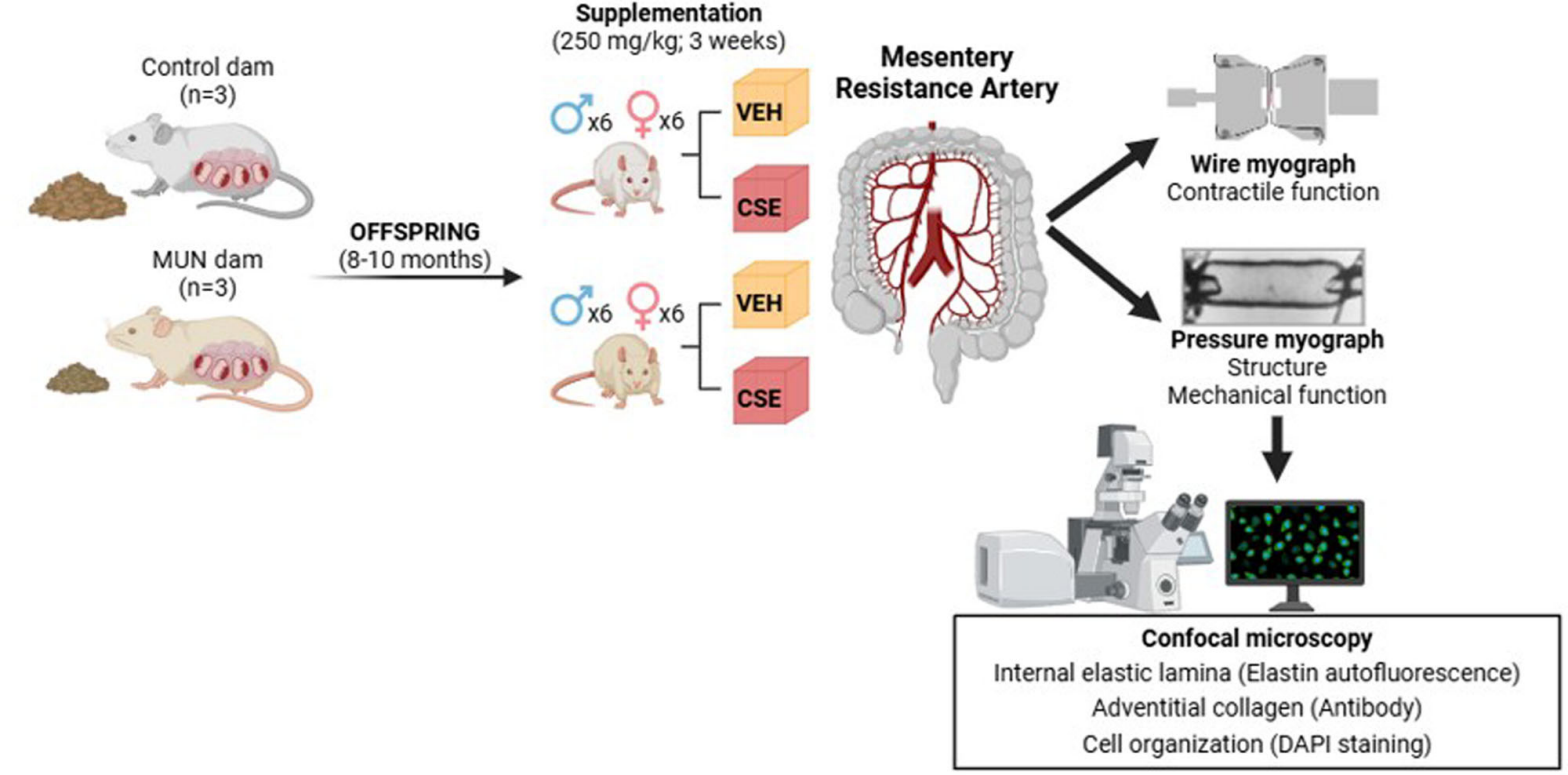
Experimental design. Abbreviations: CSE, cocoa shell extract; DAPI, 4′,6‐diamidino‐2‐phenylindole; MUN, maternal undernutrition; VEH, vehicle.

Thereafter, the whole mesentery was isolated, and second‐order branches [mesenteric resistance arteries (MRA)] were dissected and kept in cold Krebs–Henseleit solution (KHS; mM: 115 NaCl, 4.6 KCl, 1.2 MgSO_4_, 1.2 KH_2_PO_4_, 25.0 NaHCO_3_, 2.5 CaCl_2_, 0.01 EDTA and 11.0 glucose) with 95% oxygen–5% CO_2_. Some segments were used for wire myography and kept in KHS, and those used for pressure myography were transferred to 0 Ca‐KHS (same concentration, omitting calcium and adding 10 mM EGTA). At the end of the pressure myography study, the segments were pressure‐fixed at 80 mmHg and stored in 4% paraformaldehyde to evaluate cell organization, adventitial collagen and internal elastic lamina (IEL) organization by confocal microscopy. Figure 1 shows the experimental design.

#### 2.3.1 Pressure myography

Mechanical and structural properties of MRA were evaluated with a pressure myograph (Danish Myo Tech, Model P100, J.P. Trading I/S, Aarhus, Denmark). The MRA segment was placed in a 0 Ca‐KHS solution in an organ bath between two glass cannulas, secured with nylon suture. The organ bath was located on the stage of an inverted microscope (Zeiss Axiovert, Braun, Germany) coupled to a camera (Sony XC‐73CE, monochrome, Bangkok, Thailand) and a ×10 objective. Following a 15 min stabilization period at a pressure of 80 mmHg and temperature of 37°C, intraluminal pressure was set to 0 mmHg (in practice, it was set to 5 mmHg to avoid vessel collapse), and the arterial segment was exposed to increasing intraluminal pressures in steps of 20 mmHg to maximum of 140 mmHg. At each pressure, an image was obtained after a resting period of 3 min. Finally, the pressure was set to 80 mmHg, and the arterial segments were fixed at this pressure in 4% paraformaldehyde for 1 h and stored for further confocal microscopy experiments. Images were analysed with ImageJ software (https://imagej.net/software/fiji/), assessing internal diameter (Di) and external diameter (De). From these measurements, wall thickness (WT), cross‐sectional area (CSA) and wall‐to‐lumen ratio (W/L) were calculated using the following equations:

$$\begin{array}{c} \mathrm{WT}=\frac{\mathrm{De}-\mathrm{Di}}{2}\\ \mathrm{CSA}=\frac{\pi }{4}\times \left(De^{2}-Di^{2}\right)\\ W/L=\frac{\mathrm{De}-\mathrm{Di}}{2\times \mathrm{Di}} \end{array}$$

To evaluate the stiffness of each MRA segment, incremental Young's elastic modulus (*E*_inc_) was used. The value of *E*_inc_ was assessed by fitting circumferential wall strain (ԑ) and circumferential wall stress (σ) data from each segment to an exponential curve using the following equation, as previously described (Gutiérrez‐Arzapalo et al., 2020):

$$\sigma =\sigma_{\mathrm{origen}}^{\beta \times \varepsilon }$$

$$\sigma =\frac{P_{X}\times \mathrm{Di}}{2\times \mathrm{WT}}$$

where *P_x_* is the intraluminal pressure, and WT is wall thickness at this pressure (1 mmHg = 133.4 N/m^2^).

$$\varepsilon =\frac{\mathrm{Di}-D_{0}}{D_{0}}$$

where Di is the internal diameter at each pressure and D_0_ is the diameter at 5 mmHg. The β‐values were calculated from each stress–strain curve, being directly proportional to *E*_inc_, hence a measure of the degree of arterial stiffness.

The areas under the curves (AUCs) of the pressure–structural data (De, Di, WT, CSA and W/L) were also quantified for the analysis.

#### 2.3.2 Confocal microscopy

##### Staining

The pressure‐fixed segments were first washed in PBS in a 96‐well plate. Then, they were stained with rabbit anti‐collagen type I antibody (1:200; Sigma‐Aldrich Merck, Darmstadt, Germany) prepared in PBS for 3 h at room temperature, followed by two washing periods of 30 min. Thereafter, the segments were stained with the nuclear dye 4′,6‐diamidino‐2‐phenylindole (DAPI; Invitrogen, Thermo Fisher, Madrid, Spain; 1:500 from stock solution) and washed again for another 30 min. Then, a Cy3‐anti‐rabbit secondary antibody was applied (1:200, prepared in Triton–PBS solution) and left overnight at 4°C. Finally, the artery was washed twice with Triton–PBS for 30 min at room temperature. The segment was mounted in a well made of silicon spacers and filled with anti‐fading agent CITIFLUOR‐AF (Citifluor Ltd, UK).

##### Visualization and quantification

The segments were visualized with a Leica SP2 spectral confocal microscope (Leica Microsystems, Germany). To evaluate the layer thickness and cell density in the adventitia and medial layers, stacks of 1‐µm‐thick serial optical sections were obtained throughout the arterial wall in two or three different regions of the segment with the DAPI wavelength (359 nm excitation/457 nm emission) with a ×40 oil immersion objective at zoom × 2, as previously described (Gutiérrez‐Arzapalo et al., 2020). The first image of the stack was established as the image with the first visible adventitial cell nuclei, and the last was the image with the last visible smooth muscle cell (SMC) nuclei, considering the shape and orientation of the nuclei, which allow cells in the adventitia to be distinguished clearly from SMCs. Quantification was performed by FIJI free software. From each stack of images, the numbers of adventitial cells and SMCs were counted, layer thickness was determined by the number of planes showing each cell type, and layer volume was calculated as layer thickness × image area. From these data, cell density was calculated for adventitial and medial layers (number of cells/layer volume).

To evaluate collagen, two additional stacks of 1‐µm‐thick serial sections were obtained from the adventitial layer at 555 nm excitation/569 nm emission wavelength (Cy3‐fluorescent antibody to detect collagen), in identical conditions of laser intensity, brightness and contrast. For quantification, the outermost region of the adventitia (12 µm thick) was used to obtain a projection, where the amount of collagen was quantified as the mean intensity with FIJI free software.

To study the structure of IEL fenestrae, MRA segments were visualized with 488 nm excitation/500–600 nm emission, the wavelength at which the elastin is visible, based on its autofluorescence properties, as previously described (Gutiérrez‐Arzapalo et al., 2020). Stacks of 0.5‐µm‐thick serial optical sections were captured from two different regions of the IEL with a ×40 oil immersion objective at zoom × 4, in identical conditions of laser intensity, brightness and contrast. The IEL was reconstructed into a projection, where fenestrae area and image area were quantified with FIJI free software. Data were reported as relative fenestra area (area of fenestra/total image area).

#### 2.3.3 Wire myography

Two‐millimetre MRA segments were mounted between 25 µm tungsten wires in a four‐chamber wire myograph system (Multi Wire Myograph System 610M, DMT, Denmark) coupled to a PowerLab data acquisition system (AD Instruments, Castle Hill, NSW, Australia). The chamber was filled with KHS solution at 37°C and gassed with a mixture of 95% oxygen and 5% CO_2_. After a resting period of 15 min, with the wires touching each other, the vessels were gradually stretched for periods of 1 min until achieving a standard tension of ∼4 mN (equivalent to ∼50 mmHg of physiological pressure), which is the pressure estimated for MRA of rats, according to the original method of Mulvany and Halpern (1977) and recently revised (Schubert et al., 2023). Thereafter, myogenic responses to progressive stretching of MRA were recorded. After a 15 min resting period, maximal contraction was assessed with a 125 mM KCl solution until reaching a plateau in terms of force generation, followed by three KHS washing periods, until a baseline was reached. Thereafter, a concentration–response curve to KCl (from 10.7 to 125 mM) was performed. After washing and returning to baseline, a concentration–response curve to noradrenaline (NA; from 10^−8.5^ to 10^−4^ M) was obtained. To evaluate potency to the agonist, pD2 values were calculated with GraphPad Prism (v.10.0.0 for Windows, GraphPad Software, Boston, MA, USA; www.graphpad.com), and AUCs of the concentration–response curves were calculated for statistical analysis.

### 2.4 Statistical analysis

Data were analysed, including outliers and separately by sex, using the R language (https://www.R‐project.org/) with the RStudio interface (v.2023.6.0.421; IDE; https://posit.co), with the *rio*, *ggplot2*, *dplyr*, *Lme4*, *lmerTest*, *modelr*, *performance* and *CompareGroups* packages. The sample size (*n*) represents a single rat, and if more than one set of data was obtained from the same rat, they were averaged prior to the analysis. The distribution of the variables was tested by Q–Q plot. Pressure myography and wire myography data were represented as curves, and the AUC was used for statistical analysis. The confocal microscopy data were represented as box‐and‐whisker plots showing the median and interquartile range (Q1; Q3). Given that the rats used in the study (*n* = 6 per experimental group) belonged to only three different dams, to evaluate the possible litter effect on CSE actions a linear mixed‐effects model was used for statistical analysis, considering supplementation as a fixed effect and dam as a random effect. A *p*‐value of <0.05 was considered statistically significant and indicated in the figures, together with Cohen's *d* index to show the size effect.

## 3 RESULTS

### 3.1 Vascular structure and mechanical function assessed by pressure myography

**FIGURE 2:**
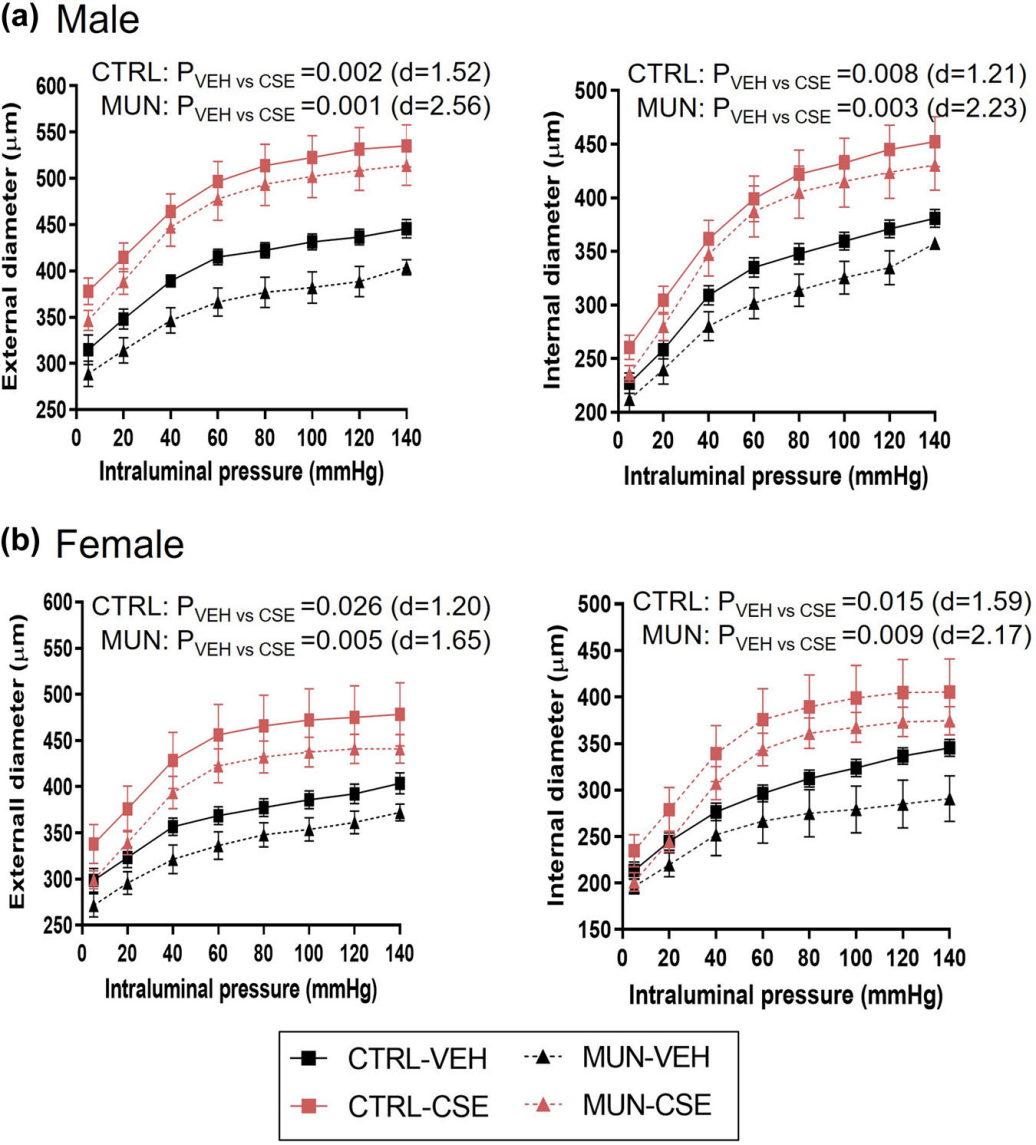
Pressure–diameter curves. Data were obtained by pressure myography in MRA from control and MUN adult rats after 3 weeks of supplementation with a neutral gelatine (VEH) or CSE. The *p*‐value and Cohen's *d* index were obtained from the AUC by a linear mixed‐effects model considering supplementation as a fixed effect and dam as a random effect. Each experimental group includes one or two segments from six rats and three different dams. Abbreviations: AUC, area under the curve; CSE, cocoa shell extract; CTRL, control; MRA, mesenteric resistance artery; MUN, maternal undernutrition; VEH, vehicle.

To elucidate changes in vascular geometry and passive mechanical behaviour, MRA was studied by pressure myography. MUN‐VEH males had reduced Di and De compared with Control‐VEH. CSE supplementation significantly increased both diameters in MUN‐CSE and Control‐CSE in comparison to the respective VEH groups, with AUC being significantly enlarged (Figure 2a). The lumen increase was 21.1% in MUN and 23.5% in control groups.

In females, similar results were found; MUN‐VEH had a reduced Di and De compared with Control‐VEH. CSE supplementation increased both parameters in MUN‐CSE and Control‐CSE rats compared with the respective VEH groups, with a 20.5% enlargement in MUN and 28.4% in the control group (Figure 2b).

**FIGURE 3:**
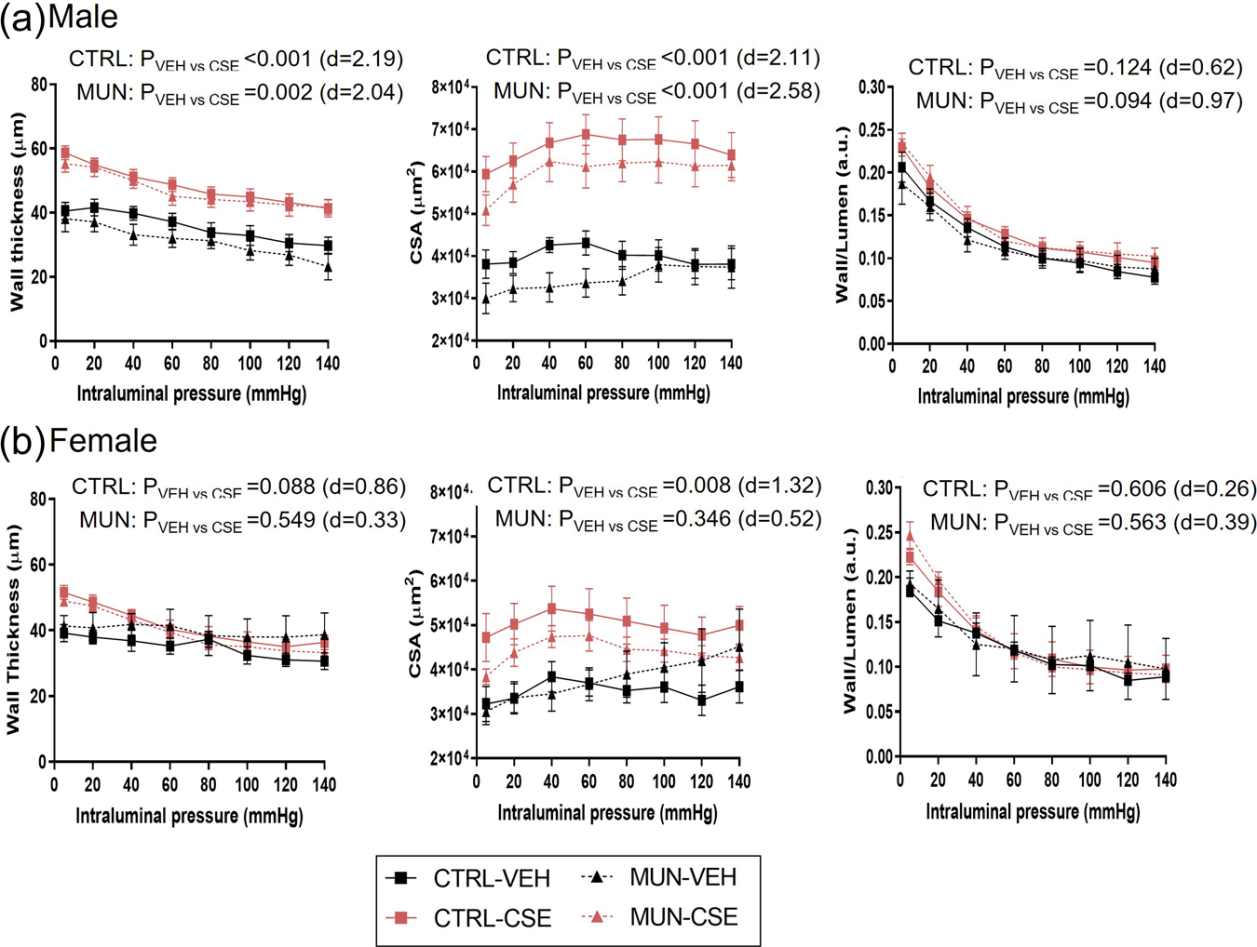
Pressure–WT, pressure–CSA and wall‐to‐lumen ratio. Data were obtained by pressure myography in MRA from control and MUN adult rats after 3 weeks of supplementation with a neutral gelatine (VEH) or CSE. The *p*‐value and Cohen's *d* index were obtained from the AUC by a linear mixed‐effects model considering supplementation as a fixed effect and dam as a random effect. Each experimental group includes one or two segments from six rats and three different dams. Abbreviations: AUC, area under the curve; CSA, cross‐sectional area; CSE, cocoa shell extract; CTRL, control; MUN, maternal undernutrition; VEH, vehicle; WT, wall thickness.

In males, MUN‐VEH had similar WT (*p* = 0.448; *d* = 0.39) and CSA compared with Control‐VEH (*p* = 0.537; *d* = 0.30). These parameters were significantly increased in CSE groups (Figure 3a,b). In MUN‐CSE, CSA enlarged by 123.4% and in Control‐CSE 68.0%. Regarding the wall‐to‐lumen ratio, no significant differences were detected between Control‐VEH and MUN‐VEH, and CSE did not influence this parameter in either group.

In MUN‐VEH females, WT (*p* = 0.405; *d* = 0.61) and CSA (*p* = 0.639; *d* = 0.24) were not significantly different from Control‐VEH. CSE supplementation significantly increased CSA in Control‐CSE (32.9%) compared with VEH counterparts. No significant differences were detected in the wall‐to‐lumen ratio between Control‐VEH and MUN‐VEH, and CSE did not influence this parameter in either group of females (Figure 3b).

**FIGURE 4:**
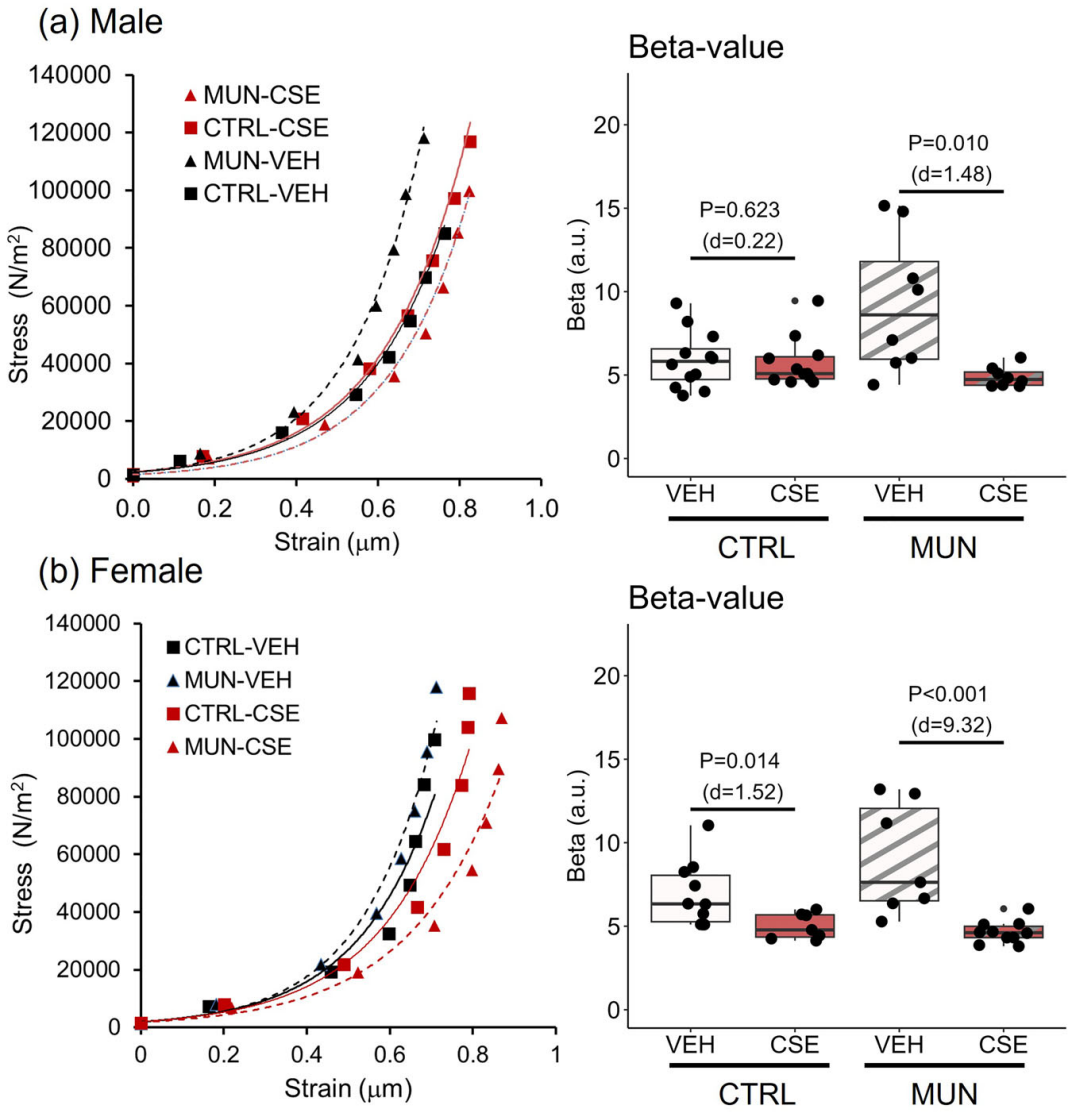
Stress–strain relationship and calculated β‐values. Data were obtained by pressure myography in MRA from control and MUN adult rats after 3 weeks of supplementation with a neutral gelatine (VEH) or CSE. Left panels show the adjusted stress–strain curves and right panels the median with interquartile range (Q1; Q3) of the β‐values obtained from them. The *p*‐value and Cohen's *d* index were obtained by a linear mixed‐effects model considering supplementation as a fixed effect and dam as a random effect. Each experimental group includes one or two segments from six rats and three different dams. Abbreviations: CSE, cocoa shell extract; CTRL, control; MRA, mesenteric resistance artery; MUN, maternal undernutrition; VEH, vehicle.

MUN‐VEH males showed a leftward shift of the stress–strain curve and significantly larger β‐value (*p* = 0.020; *d* = 1.26), indicative of increased arterial stiffness. In the MUN groups, CSE supplementation significantly reduced vessel stiffness, with a 44.9% reduction in the parameter β compared with the MUN‐VEH group. No significant differences were observed in the values reported between Control‐CSE and Control‐VEH groups (Figure 4a).

In females, no changes in stress–strain curves or the parameter β were found in MUN‐VEH compared with Control‐VEH (*p* = 0.367; *d* = 1.70). CSE supplementation shifted stress–strain curves to the right and significantly reduced β‐values in both Control‐CSE (24.6% reduction) or MUN‐CSE females (39.6% reduction), compared with the respective VEH counterparts (Figure 4b).

### 3.2 Vascular ECM and cellular components

IEL was evaluated by confocal microscopy based on the autofluorescence properties of elastin, the main component of the IEL, and the relative area of fenestrations was assessed owing to their role in elasticity in resistance arteries (Briones et al., 2003).

**FIGURE 5:**
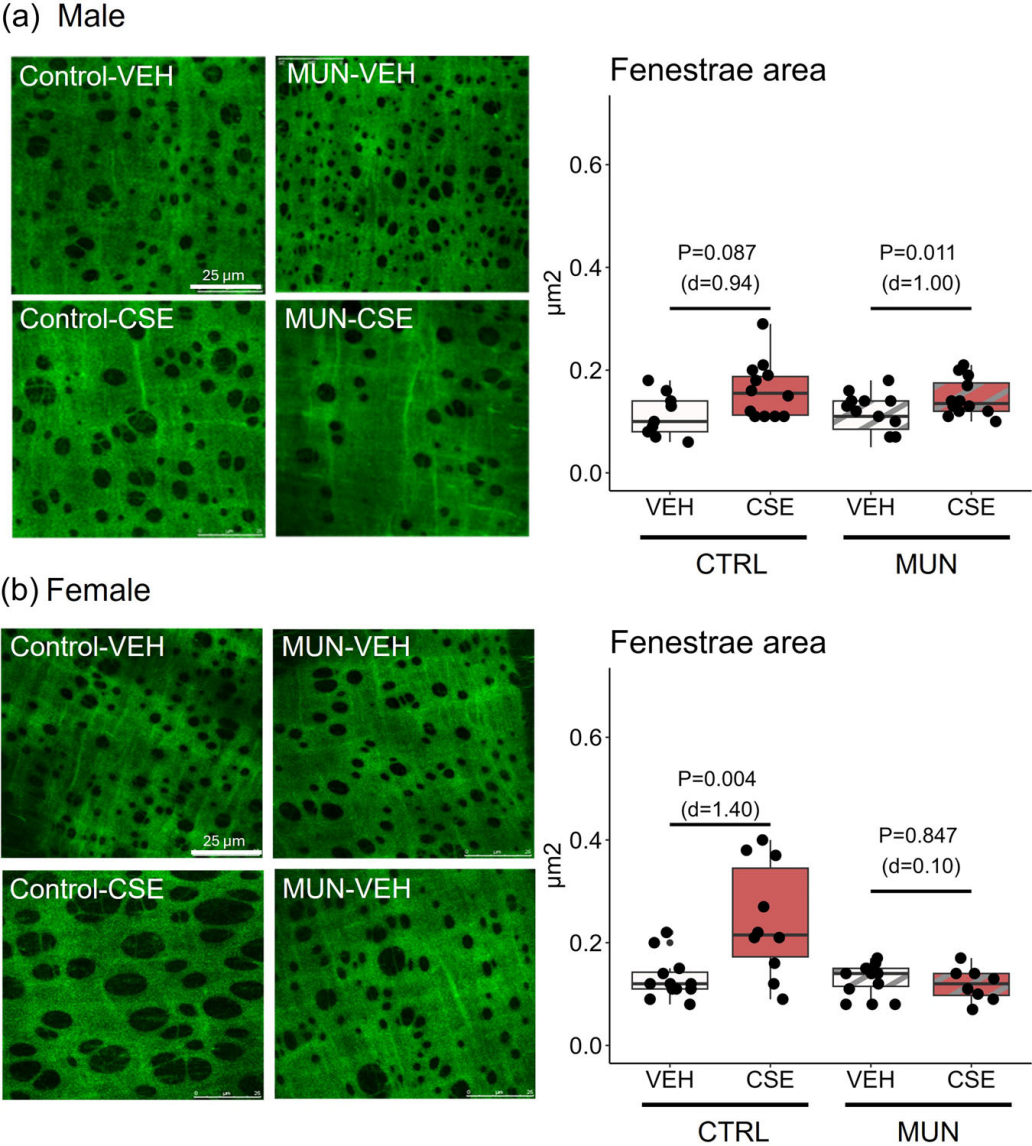
Area of fenestrae in the IEL. Data were obtained by confocal microscopy in pressure‐fixed MRA segments from control and MUN adult rats after 3 weeks of supplementation with a neutral gelatine (VEH) or CSE. The *p*‐value and Cohen's *d* index were obtained by a linear mixed‐effects model considering supplementation as a fixed effect and dam as a random effect. Each experimental group includes one or two segments from six rats and three different dams. Representative image projections were reconstructed from serial images taken with a ×40 zoom × 4 at 488 nm/500–600 nm (elastin autofluorescence). Image size 512 pixels × 512 pixels. Scale bars: 25 µm. Abbreviations: CSE, cocoa shell extract; CTRL, control; IEL, internal elastic lamina; MRA, mesenteric resistance artery; MUN, maternal undernutrition; VEH, vehicle.

In males, no differences in relative fenestra area were detected between Control‐VEH and MUN‐VEH. MUN males supplemented with CSE showed a 22.7% significant enlargement of fenestrae compared with MUN‐VEH (Figure 5a).

Regarding females, no significant differences were observed between MUN‐VEH and Control‐VEH groups. CSE supplementation did not influence fenestra area in MUN females but significantly enlarged fenestra area in a 79.2% in Control females (Figure 5b).

**FIGURE 6:**
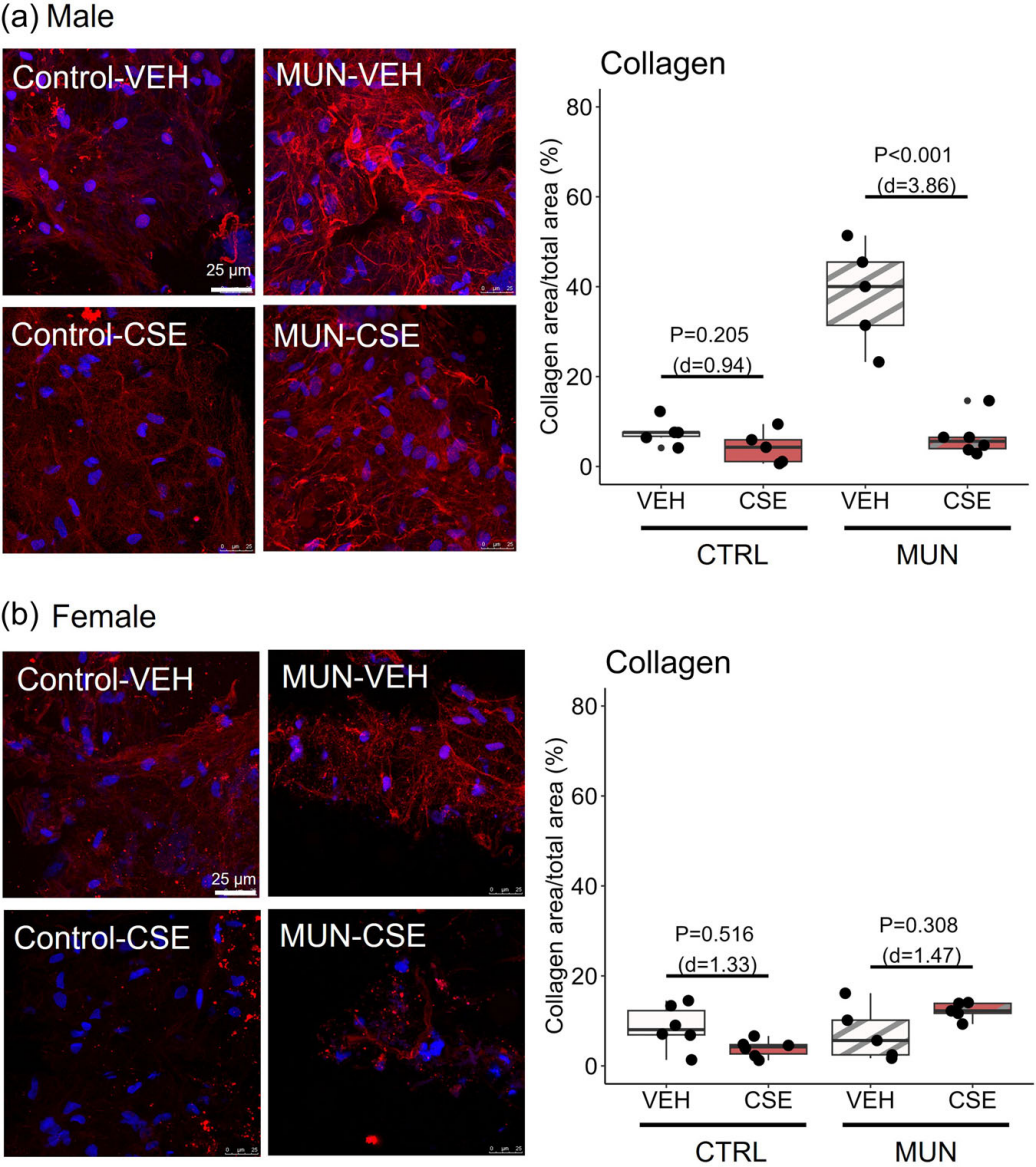
Adventitial collagen content. Data were obtained by confocal microscopy in pressure‐fixed MRA segments stained with collagen antibody, from control and MUN adult rats after 3 weeks of supplementation with a neutral gelatine (VEH) or CSE. The *p*‐value and Cohen's *d* index were obtained by a linear mixed‐effects model considering supplementation as a fixed effect and dam as a random effect. Each experimental group includes one or two segments from six rats and three different dams. Representative image projections were reconstructed from serial images taken with a ×40 zoom × 4 at 555 nm/569–600 nm (Cy3 secondary anti‐collagen antibody). Image size 512 pixels × 512 pixels. Scale bars: 25 µm. Abbreviations: CSE, cocoa shell extract; CTRL, control; MRA, mesenteric resistance artery; MUN, maternal undernutrition; VEH, vehicle.

In males, a significantly larger area of adventitial fibrillar collagen was found in MUN‐VEH versus Control‐VEH (*p* < 0.001; *d* = 3.94). CSE administration significantly reduced collagen in the MUN‐CSE group by 88.9%. No changes in collagen were found in Control‐CSE compared with Control‐VEH (Figure 6a). In females, no changes in collagen were found either in MUN‐VEH compared with Control‐VEH rats or after CSE supplementation (Figure 6b).

**FIGURE 7:**
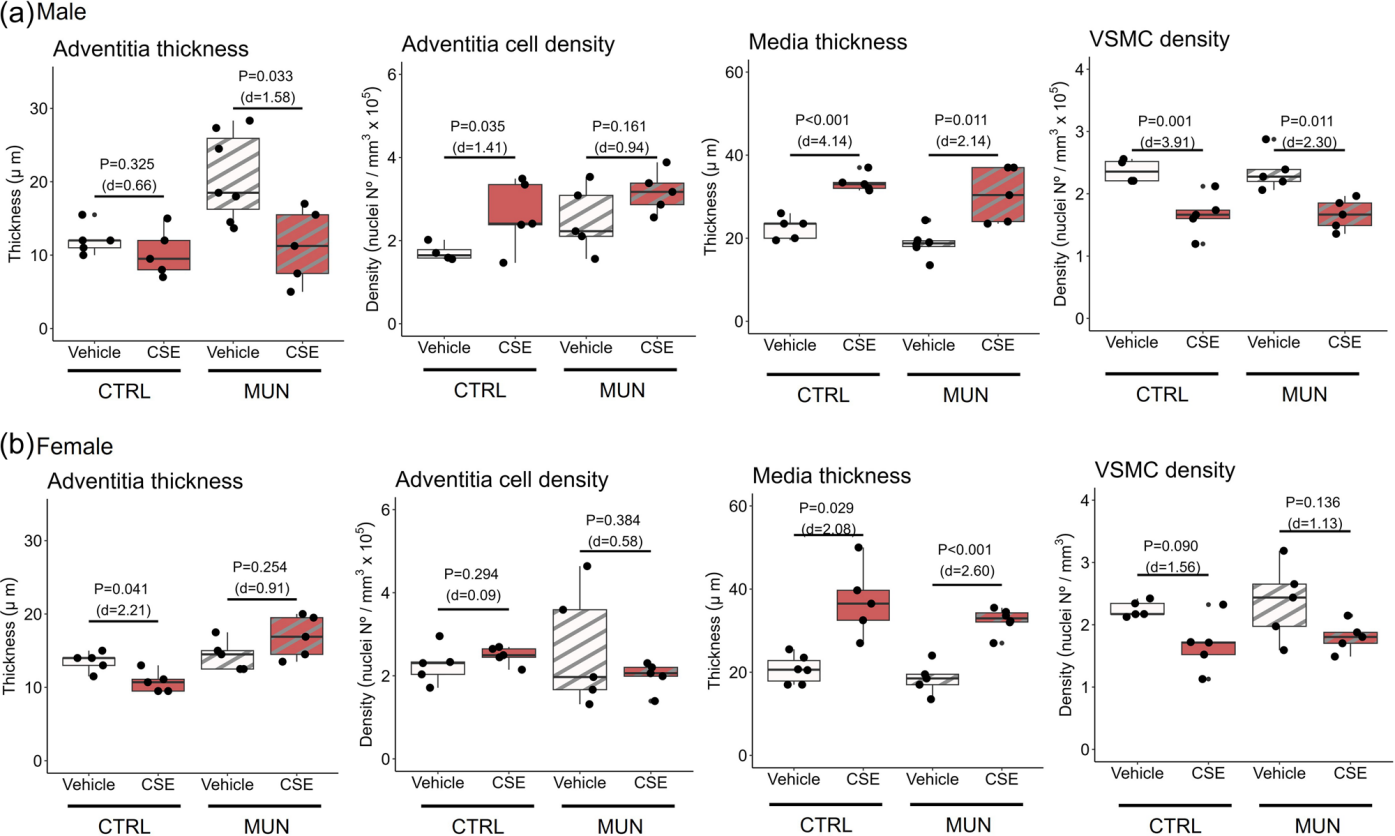
Adventitia and media layer characteristics. Data were obtained by confocal microscopy in pressure‐fixed MRA segments from control and MUN adult rats after 3‐weeks of supplementation with a neutral gelatine (VEH) or CSE. Arteries were visualized at 359 nm excitation/457 nm emission wavelengths (4′,6‐diamidino‐2‐phenylindole staining). The *p*‐value and Cohen's *d* index were obtained by a linear mixed‐effects model considering supplementation as a fixed effect and dam as a random effect. Each experimental group includes one or two segments from six rats and three different dams. Abbreviations: CSE, cocoa shell extract; CTRL, control; MRA, mesenteric resistance artery; MUN, maternal undernutrition; VSMC, vascular smooth muscle cell.

Adventitial and medial layer thickness and cell density were evaluated to assess the role of each layer in the remodelling process. In MUN‐VEH males, the adventitial layer thickness was significantly larger compared with Control‐VEH males (*p* = 0.013; *d* = 1.78), without differences in cell density. MUN‐CSE rats showed a significant reduction of adventitial thickness by 37.8%, whereas density was not modified (Figure 7a).

In male rats, media thickness tended to be smaller in MUN‐VEH compared with Control‐VEH (*p* = 0.083; *d* = 1.18), with no differences in cell density. CSE supplementation significantly enlarged medial thickness in both MUN‐CSE by 62.2% and in Control‐CSE by 38.3% compared with their respective non‐supplemented groups, significantly decreasing SMC density in MUN‐CSE (26.8%) and control‐CSE (29.2%) (Figure 7a).

In female rats, no significant differences were found in adventitia thickness or cell density between MUN‐VEH and Control‐VEH groups. In MUN females, CSE supplementation did not modify these parameters, whereas in Control females supplementation significantly reduced adventitia thickness (Figure 7b). In the medial layer, CSE supplementation significantly increased medial layer thickness in both MUN‐CSE (79.5%) and Control‐CSE (58.5%), whereas SMC density was not affected by supplementation (Figure 7b).

### 3.3 Contractile responses

**FIGURE 8:**
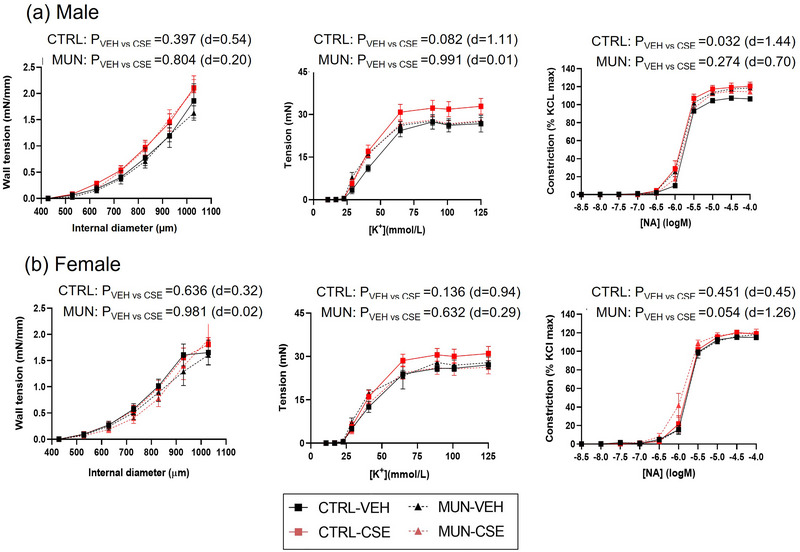
Contractile responses. Data were assessed by wire myography in control and MUN adult rats before and after 3 weeks of supplementation with a neutral gelatine (VEH) or CSE. The *p*‐value and Cohen's *d* index were obtained from the AUC by a linear mixed‐effects model considering supplementation as a fixed effect and dam as a random effect. Each experimental group includes one or two segments from six rats and three different dams. *n* = 6 rats per group. Abbreviations: AUC, area under the curve; CSE, cocoa shell extract; CTRL, control; MUN, maternal undernutrition; NA, noradrenaline; VEH, vehicle.

In males, no differences in myogenic response were detected between Control‐VEH and MUN‐VEH rats, and CSE supplementation did not have a significant effect in control or MUN groups (Figure 8a, left panels). Likewise, no significant differences in KCl‐induced contractions were found between MUN‐VEH and Control‐VEH males, and supplementation with CSE did not modify either response (Figure 8a, middle panels). In MUN‐VEH males, responses to NA were significantly elevated compared with control‐VEH (AUC: *p*‐value = 0.018; *d* = 1.64), without differences in pD2 values (Control‐VEH = 5.64 [5.62; 5.66]; MUN‐VEH = 5.69 [5.63; 5.79]; *p* = 0.887). In MUN rats, CSE supplementation did not modify the response (Figure 8a, right panels) or pD2 values (MUN‐CSE = 5.63 [5.58; 5.74]; *p*‐value with MUN‐VEH = 0.999). In control males, CSE supplementation tended to increase KCl‐induced contractions and increased NA‐induced contractions, which were significantly larger compared with Control‐VEH (Figure 8a, middle and right panels), whereas pD2 values were not significantly modified by CSE (Control‐CSE = 5.70 [5.65; 5.80]; *p*‐value with Control‐VEH = 0.582).

In females, no differences in myogenic response were found between MUN‐VEH and Control‐VEH rats, and no modifications were detected with supplementation (Figure 8b, left panels). KCl‐induced contractions were also similar between MUN‐VEH and Control‐VEH females. Supplementation with CSE did not modify the KCl response in either control or MUN females (Figure 8b, middle panels). No significant differences were found in NA responses between Control‐VEH and MUN‐VEH, either in the AUC (Figure 8b, right panels) or in the pD2 values (Control‐VEH = 5.65 [5.21; 5.72]; MUN‐VEH = 5.80 [5.65; 6.04]; *p* = 0.999). CSE supplementation did not modify NA responses or pD2 values in either MUN (MUN‐CSE = 5.80 [5.65; 6.04]; *p* = 0.329) or Control females (Control‐CSE = 5.63 [5.55; 5.74]; *p* = 0.999).

## 4 DISCUSSION

**FIGURE 9:**
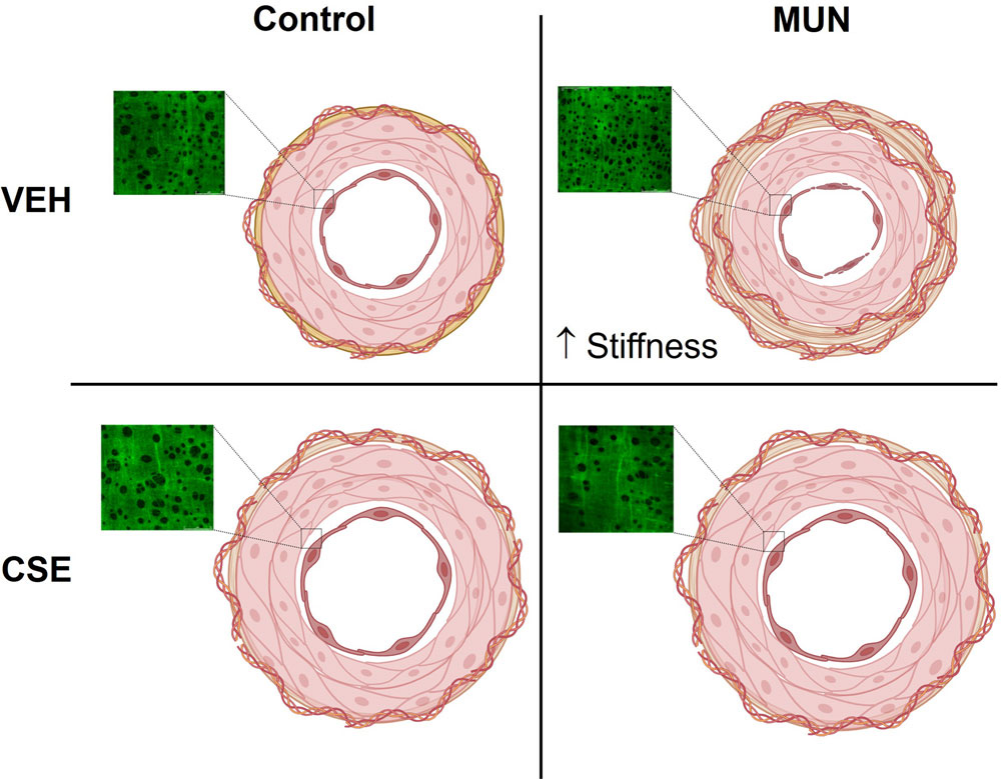
Summary of effects of CSE supplementation on mesenteric resistance artery structure and mechanical function in male rats. Mesenteric resistance artery from MUN hypertensive males exhibits inward hypotrophic remodelling and increased stiffness, related to elevated collagen content. CSE supplementation enlarged the lumen size and the medial layer through smooth muscle cell hypertrophy in both control and MUN males. In MUN hypertensive males, supplementation also improved elasticity through reduction of collagen content and increasing area of fenestrae. Abbreviations: CSE, cocoa shell extract; MUN, maternal undernutrition; VEH, vehicle.

Our previous study demonstrated that CSE supplementation was able to normalize blood pressure in MUN hypertensive males, at least in part through improvement of several vasodilatory pathways in MRA. The present work was conducted to evaluate whether the blood pressure‐lowering effects of CSE were also mediated by modification of MRA structure and mechanical function, and the relationship with cellular and ECM components. Our main findings (summarized in Figure 9) are that CSE induces outward hypertrophic remodelling in MRAs from both MUN and control rats independent of sex, maintaining the wall‐to‐lumen ratio. The structural modifications were related to a reorganization of the arterial wall, promoting SMC hypertrophy while reducing ECM, particularly adventitial collagen, which improved vascular elasticity. Given that the CSE‐induced remodelling was accompanied by a normal wall‐to‐lumen ratio and was associated with a blood pressure‐lowering effect and increased MRA vasodilatation, we suggest that these structural changes are adaptative and could be attributed to improved blood flow in the resistance vasculature.

MRAs from MUN males exhibited inward remodelling, a well‐known common feature of resistance vessels in hypertension also found in other experimental models, such as the spontaneously hypertensive rat (SHR), and in chronic kidney disease in humans (Conde et al., 2011; Mulvany, 2002; Quek et al., 2016; Schiffrin, 2015), in MRA from younger MUN rats (Gutiérrez‐Arzapalo et al., 2020) and main mesenteric artery (Vieira‐Rocha et al., 2022). The confocal data showed that the medial layer was reduced, whereas adventitial layer and collagen content increased. Given that MRA from the same animals showed impaired vasodilatation (Ruvira et al., 2024), we suggest that medial hypotrophy might be related to a long‐term reduction in blood flow, as demonstrated previously in resistance arteries in several animal models (Buus et al., 2001; Chambers et al., 2020; Stepp et al., 2004). Confocal microscopy data also revealed that SMC density was not reduced in MUN arteries, which is compatible with a possible volume reduction and dedifferentiation of SMC towards a secretory phenotype, increasing ECM production, as reported in experimentally decreased blood flow studies (Buus et al., 2001). We did not find any alteration in SMC contractility, either in myogenic tone or in depolarization by KCl. However, an increased response to NA without a change in sensitivity was observed in MUN males, also demonstrated in other models of fetal programming, including uterine vessel ligation in rats (Voggel et al., 2021), rat gestational hyperglycaemia (de Sá et al., 2017) and in lambs exposed to hypoxia (Brain et al., 2019). Increased adrenergic contraction in the above‐mentioned models has been proposed to be related to endothelial dysfunction and increased oxidative damage, rather than to α‐adrenergic receptors.

In MUN‐VEH females, inward remodelling was also observed despite a normal systolic blood pressure, as we have previously described (Gutiérrez‐Arzapalo et al., 2020), suggesting that it is not a consequence of hypertension. A possible explanation is that exposure to intrauterine stress causes early vascular structural alterations, even observed in fetuses from hypoxic pregnancies (Hansell et al., 2022). Despite similar MRA lumen reduction in MUN males and females, the latter had a largely preserved mechanical function, which, together with greater vasodilatory capacity (Ruvira et al., 2024), might contribute to the normal blood pressure observed in MUN females. Our previous findings suggest that the better adaptation of females to adverse intrauterine environments in comparison to males might be related to their larger antioxidant–oxidant balance from an early age (Rodríguez‐Rodríguez et al., 2015), given that oxidative stress is a relevant factor in vascular dysfunction and fibrosis. Other possible mechanisms implicated in the sex differences in fetal programming of hypertension are related to the sympathetic nervous system and renin–angiotensin system, being relevant the implication of sex hormones (Dasinger et al., 2016; Roghair et al., 2009).

CSE induced outward hypertrophic remodelling independent of sex or group, with preserved wall‐to‐lumen ratio, suggesting that the structural alterations are adaptive. Given that a chronic increase in blood flow induces this type of remodelling in resistance arteries (De Mey et al., 2005) and that CSE supplementation improved vasodilatation, we proposed increased flow as a potential mechanism (Ruvira et al., 2024). Besides, outward hypertrophic remodelling related to increased flow has been reported after 2 weeks of resveratrol supplementation (Petit et al., 2016), and cocoa polyphenols and methylxanthines have been demonstrated to enhance vasodilatation and blood flow in humans (Garcia et al., 2018; Grassi et al., 2015; West et al., 2014). We propose a similar mechanism, because these bioactive compounds are present in the extract, and we have evidence that they exert arterial relaxation directly, in vitro (Rodríguez‐Rodríguez et al., 2022).

MUN males also showed increased vessel stiffness, evidenced by the leftward shift of the stress–strain relationship and larger β‐values. This mechanical alteration was associated with increased adventitial thickness and collagen content, also observed in models of fetal programming induced by Zn deficiency (Mendes Garrido Abregú et al., 2018) and hypoxia (Kumar et al., 2020), among others. Collagen is the main ECM protein contributing to fibrosis. However, elastin alterations (including changes in content, fragmentation or abnormal deposition) have been demonstrated in hypertensive patients and animal models, also contributing to reduced elasticity (Arribas et al., 2006). In resistance arteries, we have demonstrated that smaller‐sized fenestrations of the IEL participate in vascular stiffness in hypertension (Briones et al., 2003) and obesity rodent models (Gil‐Ortega et al., 2016). However, in the present study this was not observed, and MRA stiffness seems to be related mainly to collagen deposition. We cannot discard alterations in other ECM components, such as fibronectin, proteoglycans or vascular calcification, which have been shown to be important particularly during the ageing process (Zhang et al., 2020). MUN females showed a better mechanical function compared with males, with normal elasticity and no alteration in ECM/cellular components. This sex difference might be related to the better oxidative status in females or to variations in ECM turnover, because adult MUN males exhibit significantly higher plasma matrix metalloproteinase (MMP)‐9 activity, whereas MUN females do not (Gutiérrez‐Arzapalo et al., 2020). CSE supplementation notably increased the fenestrae area of IEL and reduced collagen content in the adventitia, together with a reduction in β‐values. Given that fibrosis is related to oxidative stress, these effects might be related to the bioactive compounds of the extract, which exhibit a capacity to reduce reactive oxygen species production and scavenging actions. Polyphenols have well‐demonstrated antifibrotic actions in the cardiovascular system. Lentil polyphenols attenuated perivascular fibrosis in angiotensin II‐induced hypertension (Yao et al., 2012); (−)‐epicatechin treatment reduced fibrosis in a mouse model of cardiomyopathy (De Los Santos et al., 2018), and blueberry extract supplementation in SHR reduced MRA stiffness (Thandapilly et al., 2022). In humans, Heiss et al. (2015) demonstrated that supplementation with cocoa flavanols in young and old subjects decreased pulse wave velocity, a marker of arterial stiffness. Regarding the mechanism, we propose that a reduction of oxidative stress, which is implicated in fibrosis, could participate. CSE components could also have reduced transforming growth factor‐β1, which participates in fibrosis signalling pathways, as demonstrated by supplementation with cocoa bean extract in a rodent model of liver fibrosis (Budryn et al., 2018). CSE might also modify elements related to ECM degradation or turnover, because caffeine decreased MMP‐2 and MMP‐9 activity in a rat model of liver fibrosis (Arauz et al., 2014), and cocoa procyanidins inhibited the expression and activation of MMP‐2 in human vascular SMCs (Lee et al., 2008). The structural and mechanical alterations observed in the MUN model were similar to those reported in other models of hypertension; even the same type of remodelling was reported in SHR and MUN by us (Vieira‐Rocha et al., 2022). Given that oxidative stress participates in these alterations, it is expected that CSE (which has antioxidant actions) could have similar effects in other models of cardiovascular disease, supporting its health potential.

We also detected a hypertrophic effect of CSE, compatible with SMC enlargement rather than hyperplasia. We cannot discard an effect on apoptosis, which was not explored in the present study, because protocatechuic acid, one of the main components of CSE (Cañas et al., 2022), has been shown to have anti‐apoptotic effects in several tissues, including liver, kidney (Kassab et al., 2022) and cardiomyocytes, enhancing pro‐survival pathways (Deng et al., 2014). These trophic actions did not increase myogenic tone or contractile responses, and increased both endothelium‐dependent and independent vasodilatation (Ruvira et al., 2024), in agreement with the blood pressure‐reducing effects of CSE.

## 5 CONCLUSION

CSE supplementation improved the structural and mechanical alterations in MUN male hypertensive rats, through an enlargement of lumen size with trophic effects on SMC, reducing fibrosis through suppression of excess perivascular collagen deposition. The trophic response independent of rat sex or group might be related to a physiological response to increased blood flow owing to improved vasodilatation.

## AUTHOR CONTRIBUTIONS

Data curation; Formal Analysis; Investigation; Methodology; Software; Visualization, Writing—original draft: Pilar Rodríguez‐Rodríguez. Data curation; Investigation, Methodology; Writing—original draft: Santiago Ruvira. Data curation; Investigation; Methodology: Fatima Abderrahim. Data curation; Investigation; Methodology: Dolores Morales. Investigation; Methodology: Metee Iampanichakul. Investigation; Methodology: Lucía Juarez. Conceptualization; Formal Analysis; Investigation; Supervision; Validation; Software; Visualization; Writing—review & editing: David Ramiro‐Cortijo. Conceptualization; Funding acquisition; Investigation; Project administration; Resources; Supervision; Validation; Writing—review & editing: Silvia M. Arribas. All authors approved the final version of the manuscript and agree to be accountable for all aspects of the work in ensuring that questions related to the accuracy or integrity of any part of the work are appropriately investigated and resolved. All persons designated as authors qualify for authorship, and all those who qualify for authorship are listed.

## CONFLICT OF INTEREST

The authors declare no conflict of interest. The funders had no role in the design of the study; in the collection, analyses, or interpretation of data; in the writing of the manuscript; or in the decision to publish the results.

## Data Availability

The corresponding author had full access to all the data in the study and takes responsibility for the integrity of the data and the accuracy of the data analysis. The raw data supporting the conclusions of this article will be made available by the authors on request and sending will be evaluated following Spanish ethical regulations.
